# Does Subunit Composition Influence the Intermolecular Crosslinking of Fish Collagen? A Study with Hake and Blue Shark Skin Collagens

**DOI:** 10.3390/polym12081734

**Published:** 2020-08-03

**Authors:** María Blanco, Noelia Sanz, Jesús Valcarcel, Ricardo I. Pérez-Martín, Carmen G. Sotelo

**Affiliations:** 1 Grupo de Bioquímica de alimentos, Instituto de Investigaciones Marinas, Consejo Superior de Investigaciones Científicas, Eduardo Cabello, 6, 36208 Vigo, Spain; nsanz@iim.csic.es (N.S.); ricardo@iim.csic.es (R.I.P.-M.); carmen@iim.csic.es (C.G.S.); 2 Grupo de Reciclado y Valorización (REVAL), Instituto de Investigaciones Marinas, Consejo Superior de Investigaciones Científicas, Eduardo Cabello, 6, 36208 Vigo, Spain; jvalcarcel@iim.csic.es

**Keywords:** collagen, hake, blue shark, gel permeation chromatography, light scattering, molecular weight

## Abstract

Acid-soluble collagens from European hake and Blue shark skin were isolated, characterized, and compared. As the structure of collagen determines its function, the final objective of this study was to investigate biochemical differences between both collagens to identify future potential applications. Chromatographic behavior revealed differences in collagen from both species. Increases of temperature and stirring time produced no effect on European hake collagen solubility in the mobile phase, resulting in the same chromatographic profiles. Conversely, the application of temperature and stirring-time increments showed a positive effect on Blue shark collagen solubility, resulting in different chromatographic profiles and observing higher molecular weight components when sample is incubated at 50 °C (15 min) after 48 h stirring. To test if the different chromatographic behavior exhibited by both collagens could be influenced by differences in subunit composition (alpha-chains), cation exchange chromatography was employed to separate collagen subunits. The electrophoretic patterns and gel permeation chromatography with light-scattering detection (GPC-LS) results of the obtained cation exchange peak fractions revealed differences regarding subunit composition between both species, influencing the crosslinking pattern. This is the first comparative study using GPC-LS to provide information of European hake and Blue shark collagen subunit composition.

## 1. Introduction

The use of marine collagens is experiencing an increasing tendency as an alternative to mammalian counterparts for cosmetics, tissue engineering, and other biomedical and pharmaceutical uses due to safety reasons and ethical or religious constraints [1,2,3]. Recent investigations have demonstrated that fish discards and by-products are excellent and sustainable sources of valuable biocompounds such as collagen [4,5]. Collagen is the main structural protein in connective tissue and has a particular heterotrimeric structure that varies among tissues, contributing to unique physiological functions [1,6,7]. A wide variety of collagens have been described in vertebrates, including more than 28 different types. Despite their differences, all present tropocollagen as a building block, a monomer composed of three left-handed polypeptide chains that twist together to form a right-handed helix of around 300 kDa [8,9]. The tropocollagen molecule has the capacity to self-assemble to form supramolecular aggregates conferring important structural functions ranging from mechanical properties and other biological functions (thermal stability, mechanical strength, etc.) to the final assembly of fiber network. Collagen type I is the most common form of collagen, it is a fibrillary collagen that exists fundamentally in the skin and other connective tissues as a heterotrimer of two α1-chains and one α2-chain. The length, amino acid composition or molecular weight (MW) of the α-chains have a key influence in the fibril formation and therefore in the functionality of the tissue where collagen is present [10]. Several studies have addressed the study of the structure and assembly of different collagen types by characterizing their subunit composition [11,12,13,14], which is essential to understand their properties and potential uses. Traditionally, most studies isolating and characterizing collagen from different sources have used basic electrophoretic techniques to provide information regarding the MW of their subunits [15,16,17]. However, most recent studies combine electrophoretic and chromatographic techniques to provide an in-depth analysis of the subunit composition of collagen [12,18]. Gel permeation chromatography (GPC) with light-scattering detection is a powerful method for analyzing MW of polymers in solution [19], however, its application for determining the MW of collagen is still scarce [20].

Collagen from both teleosts and elasmobranchs has been documented previously. Major differences among them were observed in sodium dodecyl sulfate–polyacrylamide gel electrophoresis (SDS-PAGE) profiles where elasmobranch collagens showed strong α1-chains while α2-chains were very faint [7]. Shark collagen from Blue shark (*Prionace glauca*) has been successfully used for the development of biomaterials showing good mechanical properties while allowing the adequate growing of bone or cartilage cells [8,21]. The use of collagen from teleosts such as cod (*Gadus morhua*) or European hake (*Merluccius merluccius*) has also been reported [9,22]. However, although evident differences were observed in functionality and apparent subunit structure, no studies exist comparing the subunit composition of both major fish types. In this study, a teleost and an elasmobranch species were selected to compare their skin collagen subunits. To the best of our knowledge, this is the first attempt to understand differences between the subunit composition of *M. merluccius* and *P. glauca* acid-soluble collagen (ASC) using cation-exchange chromatography (cIEX) and to study the MW of collagen and their subunits (alpha chains) using gel permeation chromatography with light-scattering detection (GPC-LS).

## 2. Materials and Methods

### 2.1. Extraction of Acid Soluble Collagen

**Figure 1:**
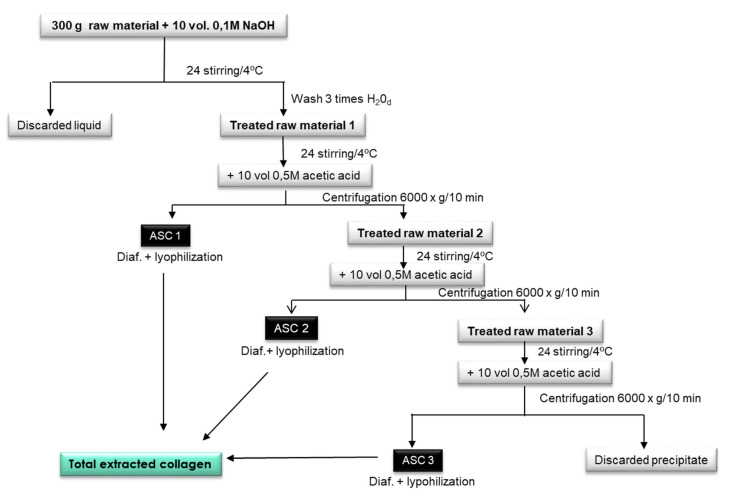
Acid-soluble collagen (ASC) extraction stages developed using *P. glauca* and *M. merluccius* raw material.

Collagen was acid-extracted from *P. glauca* skin and *M. merluccius* skin and bones according to the methodology of Hwang et al. [23] with modifications (Figure 1). Briefly, 300 g of raw material were mixed with 10 volumes of 0.1 M NaOH and stirred in a cold room (4 °C) for 24 h. The liquid phase was discarded and the treated raw material 1 was washed with distilled water until neutral pH was achieved. The washed residue was stirred for 24 h with 10 volumes of 0.5 M acetic acid, then the extract was centrifuged (3000× *g*, 15 min) and the supernatant was diafiltrated using spiral polyethersulfone membranes of 0.56 m2 (Prep/Scale-TFF, Millipore Corporation, Bedford, MA, USA) with 30-kDa MW cut-offs (MWCO). Diafiltrated solutions were then freeze-dried obtaining acid-soluble collagen (ASC); in this case, ASC1. Treated raw material 2, the residue obtained after the previous centrifugation step, was mixed with 10 volumes of 0.5 M acetic acid for 24 h and centrifuged (3000× *g*, 15 min) obtaining ASC2 (after diafiltration and freeze-drying steps, as previously described). In the case of *P. glauca* extraction, treated raw material 3, the residue obtained after the previous centrifugation step, was mixed with 10 volumes of 0.5 M acetic acid for 24 h and centrifuged (3000× *g*, 15 min) obtaining ASC3 (after diafiltration and freeze drying step, as previously described). For *M. merluccius* extraction, this third last step of re-extraction was not necessary as the treated raw material 3 was inexistent. Total extracted collagen was obtained by mixing ASC1, ASC2, and ASC3 for *P. glauca* and by mixing ASC1 and ASC2 for *M. merluccius*. This procedure was done several times, using the skin of different individuals until we had enough collagen material, which allowed us to obtain a homogenized collagen material to use in all experiments.

### 2.2. Characterization of ASC

#### 2.2.1. SDS-PAGE

The protein pattern of both ASC and cation exchange peaks collagen fractions from *P. glauca* and *M. merluccius* collagen samples was analyzed using SDS-PAGE according to the procedure described by Laemmli [24]. Samples (1 mg/mL) were prepared in sample buffer containing 10.52% glycerol, 21% sodium dodecyl sulfate (SDS) (10%), 0.63% dithiothreitol (DTT), and 0.5-M Tris-HCl (pH 6.8) and heated for 5 min at 100 °C. An aliquot (8 µL) of this mixture was applied to each well in 7% SDS polyacrylamide separating gels (100 mm × 750 mm × 0.75 mm) and subjected to electrophoresis at 20 mA using a Mini-Protean II cell (Bio-Rad, Hercules, CA, USA). Afterwards, electrophoresis gels were stained with 0.04% Coomassie Blue in 25% ethanol (*v*/*v*) and 8% acetic acid (*v*/*v*) for 2 h. Excess stain was removed with several washes of destaining solution (25% ethanol (*v*/*v*), 8% acetic acid (*v*/*v*)). MWs of collagen subunits were estimated using a wide range MW standard from Amresco; additionally, a type I collagen standard from Sigma was also employed.

#### 2.2.2. Molecular Weight Determination of Collagen by GPC-LS

GPC analyses were carried out on an Agilent 1260 HPLC consisting of a G1311B quaternary pump, a G1329B injector, a G1316A column oven, a G1362A refractive index detector (RID) and a dual-angle static light-scattering (DALS) G7800A detector. Four columns were used for chromatographic separations (PSS, Mainz, Germany): Proteema precolumn (5 µm, 8×, 50 mm), Proteema 1000 Å (5 µm, 8×, 300 nm), Proteema 300 Å (5 µm, 8×, 300 nm), and a Proteema 100 Å (5 µm, 8×, 300 nm). Lyophilized samples of extracted collagen from *P. glauca* and *M. merluccius* (1 mg/mL) were dissolved in the mobile phase (0.15 M sodium acetate, 0.2 M acetic acid, pH 4.5), after temperature/time treatments were centrifuged at 35,000 rpm for 15 min and filtered through a Polytetrafluoroethylene (PTFE) 0.2 µm membrane filter. A volume of 100 µL of the filtered solution was injected and analyzed at a flow rate of 0.5 mL/min with an isocratic elution profile. The peaks resultant from the ion-exchange chromatography were directly injected (100 µL) using the same conditions as lyophilized samples. Column oven was kept at 20 °C, refractive index detector (RID) at 35 °C, and DALS at 30 °C. DALS detector was calibrated with a polyethylene oxide standard (PEO) (PSS, Mainz, Germany) of 106 kDa (Mp) and polydispersity index of 1.05. Refractive index increments (dn/dc) were adopted from Meyers and Morgenstern [20], Theisen et al. [25] and Nomura et al. [26]. Data were analyzed using Agilent GPC/SEC software A.02.01.

##### Temperature and Stirring Time Effect on Collagen GPC Mobile Phase Solubility

In order to study differences on the solubility observed for *P. glauca* and *M. merluccius* collagen in the mobile phase used in GPC, three batches of 50 mL of 1 mg/mL solution of lyophilized collagen of each species were mixed with the mobile phase and stirred for different stirring times: 24 h, 48 h, 72 h, and 96 h. To test the effect of temperature on collagen solubility, at the end of each stirring time (24 h, 48 h, 72 h, or 96 h), 1.5 mL aliquots (per triplicate) were obtained and treated at 42 °C for 15 min, other 1.5 mL aliquots at 50 °C for 3 min, and other 1.5 mL aliquots at 50 °C for 15 min. In addition, an aliquot of 1.5 mL (per triplicate) was obtained and used as the control for no temperature applied. The treated samples were subjected to GPC measurements to analyze differences in the MW between the collagen of both species.

#### 2.2.3. Cation-Exchange Chromatography (cIEX)

The separation of collagen components was developed using an Agilent 1260 HPLC system consisting of a G1312A binary pump, a G1328B manual injector, a G1316A column oven, and a G1314B UV-vis variable wavelength detector. For chromatographic separations a Mono-S HR 5/5 column (Pharmacia Biotech) was used. For sample preparation, lyophilized collagen samples were dissolved in the mobile phase A (0.02 M Sodium formate, 0.07 M Sodium chloride, 2 M Urea, pH 3.8) (5 mg/mL) and left for 24 h with constant stirring. Then, samples were incubated at 50 °C for 15 min, centrifuged at 3500 rpm for 15 min, and the supernatant was filtered through polyvinylidene fluoride (PVDF) 0.22-µm membrane filter. A total volume of 100 µL of the filtered solution was injected and chromatographed at an eluent flow rate of 0.8 mL/min and 25 °C using a gradient elution profile with a binary mixture of mobile phase A and mobile phase B (0.02 M Sodium formate, 1 M Sodium chloride, 2 M Urea, pH 3.8) as follows: 100% A (0–2 min), 80% A (8–12 min), 75% A (18–21 min), 70% A (23–27 min), 60% A (27–29 min), 50% A (29–31 min), and 100% A (31–33 min). Eluents were monitored at 230 nm. All peaks were collected and ultrafiltrated using a 10-kDa MW cut-off Amicon Ultra Device. Aliquots of 100 µL of each peak were reinjected to check the purity. The rest of the volume was used for GPC to analyze the MW of those peaks.

## 3. Results

### 3.1. SDS-PAGE

**Figure 2:**
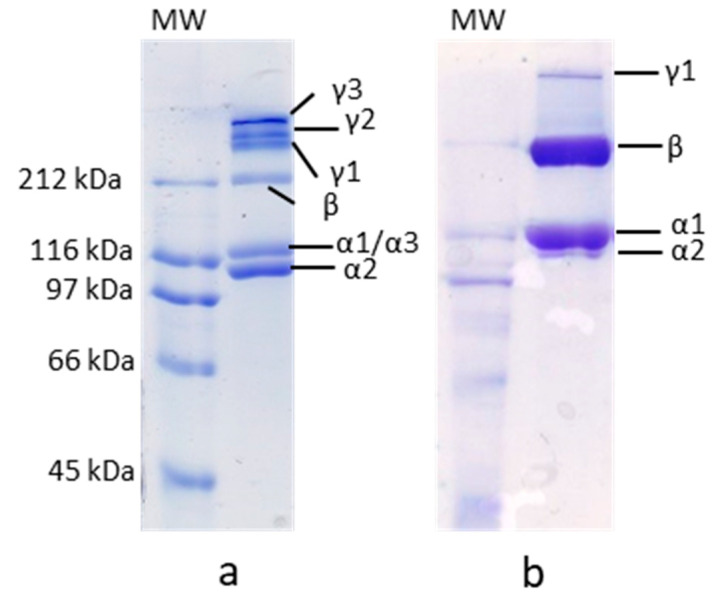
7% sodium dodecyl sulfate–polyacrylamide gel electrophoresis (SDS-PAGE) of *M. merluccius* (**a**) and *P. glauca* (**b**) collagen. 8-µL sample was loaded into each well. MW—molecular weight.

Different protein patterns were observed in 7% SDS-PAGE between *M. merluccius* and *P. glauca* ASC (Figure 2a,b, respectively). Although both collagens comprised α, β, and γ components, the proportion of them in each species was different. *M. merluccius* ASC consisted of two bands of about 120 and 100 kDa, with similar band intensities, a faint β component (dimer) with a MW of approximately 212 kDa and three γ-components (trimers). Similar results were previously reported for other teleost species [7,27]. There are studies concluding that the most common formula of γ-component in skin collagen is γ112 [28]. The existence of an α3-chain has been previously documented, with a wide distribution in Type I collagen from teleost fish, which comigrate with the α1-chain in electrophoresis [29], and that could partially explain the occurrence of those three different crosslinked γ-components (γ1, γ2, and γ3) [30]. A different protein pattern was observed for *P. glauca* ASC, which also consisted of two α-chains with MW of about 120 and 100 kDa, however, their band intensities were different than those of *M. merluccius*, the α1-chain being more than two-fold more dense than the α2-chain (Figure 2b). The presence of a faint α2-chain band together with a significantly dense β-component suggest a strong dimerization of the α2-chain into β-component to form β22-dimer. This evidence has been previously observed for other chondrichthyes [12,22,26,31]. Besides, only one γ-component was found in *P. glauca* collagen compared to *M. merluccius* (Figure 2b).

### 3.2. Molecular Weight Determination of Collagen by GPC-LS

**Figure 3:**
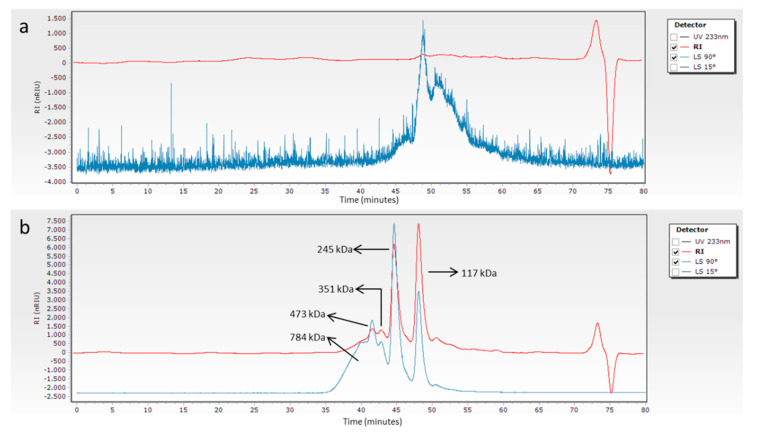

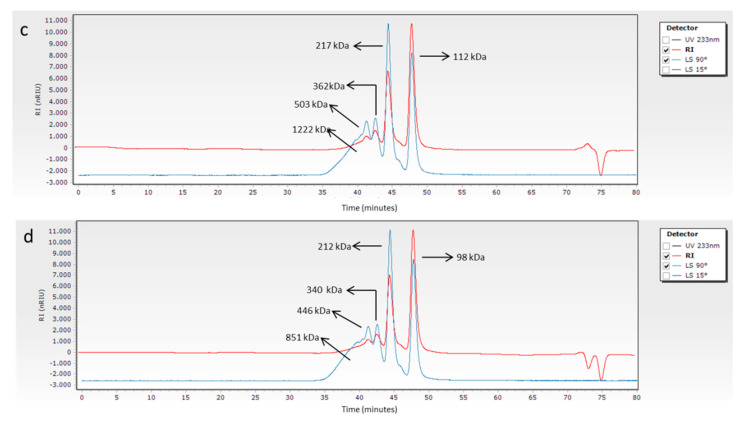
Gel permeation chromatograms and the corresponding MW of *P. glauca* ASC dissolved in mobile phase, stirred for 24 h without temperature treatment (**a**) and treated at 50 °C for 15 min after 24 h of stirring (**b**). Gel permeation chromatograms and the corresponding MW of *M. merluccius* ASC dissolved in mobile phase, stirred for 24 h without temperature treatment (**c**) and treated at 50 °C for 15 min after 24 h of stirring (**d**).

To provide an in-depth and innovative analysis of the MW of *M. merluccius* and *P. glauca* collagen, GPC using light scattering as detector (GPC-LS) was employed. The dissolution of lyophilized *P. glauca* collagen in the GPC mobile phase (0.15-M sodium acetate, 0.2-M acetic acid, pH 4.5) presented problems, which resulted in very poor and even an absent refraction index (RI) and light scattering (LS) signals, respectively, and therefore, the impossibility of determining the MW of the components of the sample (Figure 3a). However, no problems of solubility were found for *M. merluccius* ASC, which resulted in good RI and LS signals and a very good reproducibility in all conditions assayed (Figure 3c,d and Figure S1). To understand these solubility differences between collagens from *P. glauca* and *M. merluccius*, the effect of temperature/stirring-time variation on collagen solubility was studied. The stability of the collagen, and therefore its solubility, depends on the formation of stable intermolecular crosslinks [32]. Consequently, when heated above its denaturation temperature, the collagen helix would disintegrate, releasing individual α-chains differently depending on the number of intra/intermolecular crosslinks, allowing differences in solubilization.

The collagen solubility of *M. merluccius* seems not to be influenced by temperature and stirring time (Figure 3c,d and Figure S1), as the resulting chromatogram profiles after the incubation of collagen at different temperature and stirring times were very similar to those obtained without a temperature incubation step. The corresponding chromatograms of all tested conditions showed two clearly distinct peaks at elution times of 44 and 47 min corresponding to β component (dimer) and α-chains respectively. The γ-component (trimer) and higher MW components (usually not seen in separating polyacrylamide gel electrophoresis) were also present with lower concentration values as indicated by lower RI signals. Contrary to *M. merluccius* results, the application of temperature and stirring-time increments showed a positive effect on the solubility of *P. glauca* collagen (Figure 3a,b and Figure S2). When no temperature and stirring-time increments were applied, *P. glauca* collagen chromatograms lacks RI and LS signals, making it impossible to determine the MW of its components/subunits (Figure 3a). However, the application of temperature/stirring-time increments resulted in the observation of two clearly distinct peaks at elution times of 43 and 47 min, corresponding to β component (dimer) and α1,α2-chains, respectively (Figure 3b and Figure S2). The γ-component (trimer) and higher MW components were also present with lower concentration values, as indicated by low RI signals. 

**Figure 4:**
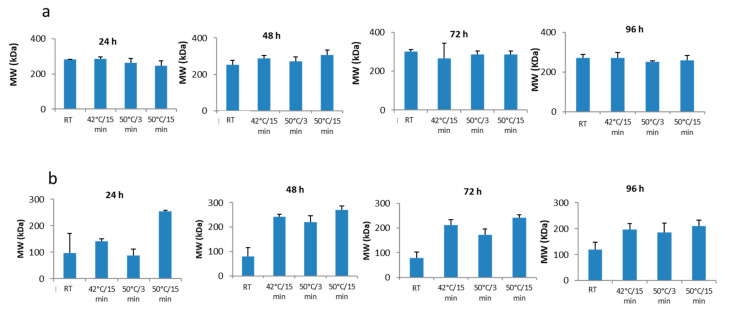
MW mean ± SD of *M. merluccius* (**a**) and *P. glauca* (**b**) collagen samples (dissolved in gel permeation chromatography mobile phase) treated at different temperature and stirring-time conditions. Bars represent mean MW from all peaks obtained in each temperature/stirring condition assayed (triplicates). RT: Room temperature.

In order to investigate the cause of these solubility differences of collagen from both species in the mobile phase, the mean MWs data obtained with GPC-LS of the collagen treated under different temperature/stirring-time conditions were analyzed and compared (Figure 4). Figure 4a shows the results for *M. merluccius*. A very similar mean MW between all conditions assayed was observed, including the control (no temperature increment from room temperature (RT) and stirring time), indicating that there is no influence of these factors on *M. merluccius* collagen solubility in mobile phase. This finding corroborates the conclusion obtained after the analysis of the GPC-LS profiles. The results obtained for *P. glauca* collagen (Figure 4b) were different, as already pointed out. The analysis of mean MW of *P. glauca* collagen, treated under different temperature and stirring times, resulted in differences between treatments. A positive effect of higher temperature on the solubilization of high MW components is observed in all stirring times analyzed, and therefore, an increase of the mean MW for this species. Furthermore, no effect of stirring-time increments is observed on the solubility of collagen if no temperature is applied. Besides, higher MW components are observed when the sample is left stirring for 48 h and just after a temperature of 50 °C is applied for 15 min. Finally, higher MW components are obtained after 48 of stirring time in the three temperatures/incubation times assayed.

These results might be in relation with differences in the intra- and intermolecular crosslinks of collagen from both species derived from differences in the subunit composition; the possible presence of an α3-chain in *M. merluccius* and a high ability of α2-chain in *P. glauca* to dimerize by covalent intermolecular bonds into β-component to form the stable β22-dimer. The level of intermolecular cross-linking plays a fundamental role in the stabilization of the fiber structure, influencing many properties of collagen [10,11,32,33]. Thus, the presence of this intermolecular β22-dimer probably contributes significantly to the higher stability of *P. glauca* collagen, compared to *M. merluccius* collagen, therefore needing a higher temperature/stirring-time conditions for its solubilization. 

### 3.3. Isolation of Collagen Components

**Figure 5:**
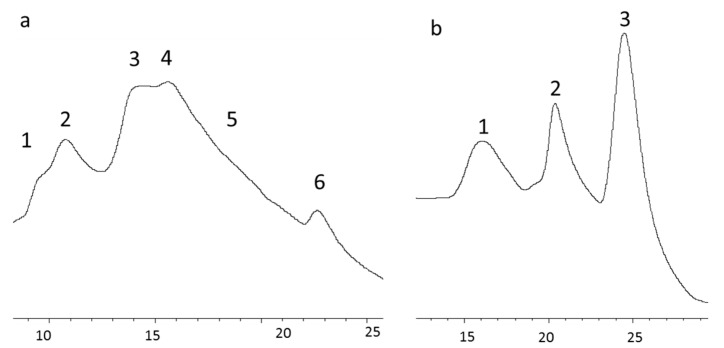
Separation of collagen type I α-chains of *M. merluccius* (**a**) and *P. glauca* (**b**) by cation-exchange chromatography (cIEX) on a Mono-S HR 5/5 column. The fractions indicated by numbers were examined by SDS-PAGE and GPC-LS. The Y-axis represents wavelength at 230 nm (mAU) while the X-axis represents time of elution.

**Figure 6:**
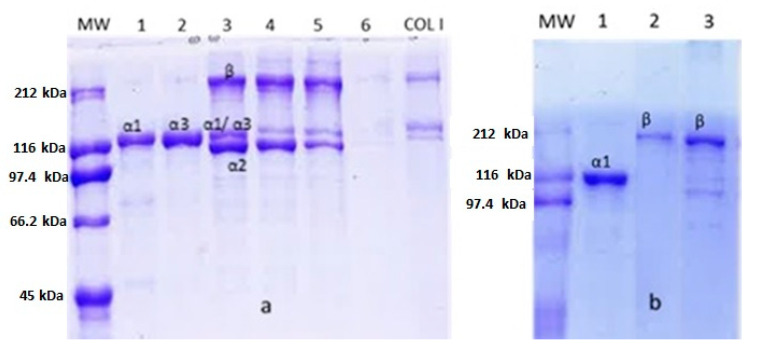
7% Sodium dodecyl sulfate poly-acrylamide gel electrophoresis (SDS-PAGE) profiles of cation-exchange peaks 1, 2, 3, 4, 5, and 6 from *M. merluccius* collagen (**a**) and cation-exchange peaks 1, 2, and 3 from *P. glauca* collagen (**b**). MW: Molecular weight marker. COL I: Type I collagen marker. 8 µL of sample was loaded into each well.

**Figure 7:**
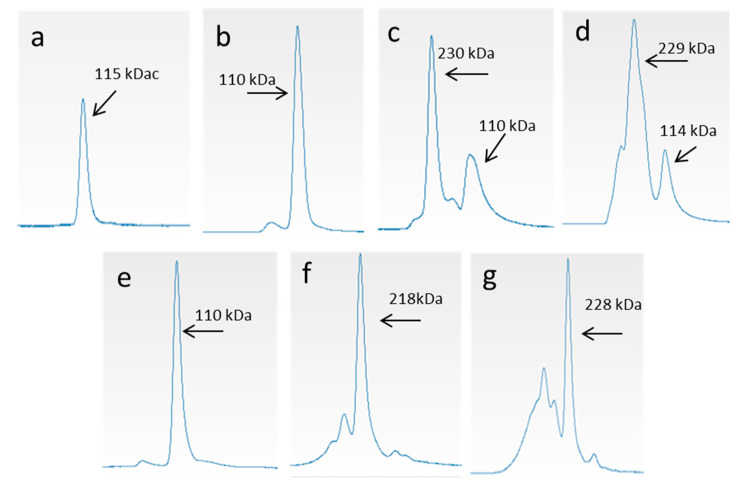
Gel permeation chromatography of *M. merluccius* (**a**–**d**) and *P. glauca* (**e**–**g**) collagen fractions collected in cIEX. For *M. merluccius* collagen, only peaks 1, 2, 3, and 4 were subjected to GPC, as peak 5 was practically equal to peak 4 due to a very low concentration of peak 6. The Y-axis represents intensity of light-scattering signal (mAU) while the X-axis represent time of elution (min).

To provide an in-depth analysis of the subunit composition of collagen from both species and to compare them, cIEX chromatography was employed. The selected chromatographic fractions, indicated by different numbers (Figure 5), were then subjected to SDS-PAGE (Figure 6) and GPC-LS (Figure 7) to study subunit composition differences between collagen species and their MWs. The chromatography elution profiles by cIEX revealed differences between skin collagens of *M. merluccius* and *P. glauca* (Figure 5a,b). *M. merluccius* collagen eluted as 5 unresolved peaks. Fractions 1 and 2 were obtained from the first peak eluting at 10 min and from the second and higher peak, respectively (Figure 5a), both peaks were not well resolved. The SDS-PAGE profile and GPC-LS of these two fractions revealed very close MWs of 115 kDa and 110 kDa (Figure 6a lanes 1 and 2 and Figure 7a,b). The existence of an α3-chain in Type I collagen in several species of teleosts has been previously reported. This α3-chain presented a very similar MW to that of the α1-chain [29], and that elutes in IEX chromatography usually after α1- and before α2-chain [13]. These finding are similar to results presented here, those first two peaks (1 and 2, in Figure 5) might correspond to α1- and α3-chain, respectively. The SDS-PAGE results of the other *M. merluccius* cIEX collagen eluted peaks (3, 4, 5, and 6), revealed that they were composed of a mixture of α1-, α2-, and α3-chains together with the β component (Figure 6a, lanes 3, 4, 5, and 6). While the intensity of the β component in peaks 3, 4, and 5 is similar, with a similar MW, determined by GPC-LS, of about 230 kDa (Figure 7c,d), the intensity of the α-chains decreased, suggesting the possible presence of different cross-linked dimers in the β component. SDS-PAGE profile of peak 3 showed similar α1/α3 intensity bands compared to α2 (Figure 6), however peak 4 and its trailing edge, identified as peak 5, contained decreasing intensities of α1/α3-chains compared to α2-chains. These results could be explained in relation to differences in the hydrophobicity of different α-chains, as it has been previously demonstrated that the content of basic amino acids influences the order of elution of alpha chains [13].

In contrast, the chromatographic profile of *P. glauca* collagen eluted as 3 clear peaks (Figure 5b). A first peak resolved at minute 16, containing the α1-chain with a MW of about 110 kDa as observed in SDS-PAGE (Figure 6b) and GPC-LS (Figure 7e). Peaks 2 and 3, resolved at minutes 21 and 24, respectively, with MWs determined by SDS-PAGE and GPC-LS of 218 kDa and 228 kDa, respectively (Figure 7f,g), corresponded with different intra/intermolecular crosslinked β-components. Under these chromatographic conditions, the α2-chain from *P. glauca* could not be separated from the β22-dimmer suggesting that it contributes to a high intermolecular and more stable crosslinking in *P. glauca* type I collagen [34]. Miles et al. [10] proposed that the higher hydrophobicity of α2-chain plays a fundamental role in the stabilization of the type I collagen due to the presence of more hydrophobic interactions between the heterotrimeric molecules, reducing the water content and increasing the binding of molecules in the fiber. 

The observations in this study reveal the presence of an α3-chain in *M. merluccius* collagen with very similar MW (determined by SDS-PAGE of cIEX peaks and also by GPC-LS) to α1-chain but with a different chromatographic behavior on cIEX chromatography, due to different hydrophobicity [35]. Besides, the SDS-PAGE profiles of the cation exchange components of *P. glauca* revealed the presence of an extensively crosslinked α2-chain which could not be separated under these chromatographic conditions.

## 4. Conclusions

This study presents evidence, based on chromatographic and electrophoretic analysis, on the differences in subunit composition of collagen from *M. merluccius* and *P. glauca*. Our results showed the presence of an α3-chain in the former and an extensive dimerization of α2-chain in the latter that might be determinant of crosslinking pattern differences between both species. We hypothesize that the observed crosslinking dissimilarities might be responsible for the different collagen solubility behavior observed in the mobile phase of GPC-LS. The higher intermolecular crosslinking observed in *P. glauca* collagen implies the need of different temperature/stirring-time conditions for its solubilization compared to *M. merluccius* collagen. The extensive dimerization of the α-2 chain found in *P. glauca* collagen, leading to high number of covalent intermolecular crosslinks favoring the process of fibril assembly, might be the responsible for its higher insolubility in mobile phase compared to *M. merluccius* collagen. This fact might contribute ultimately to the differences in mechanical stability and strength of the collagen fibrils in these species.

The observations in this study reveal the presence of an α3-chain in *M. merluccius* collagen with very similar MW (determined by SDS-PAGE of cIEX peaks and also by GPC-LS) to α1-chain but with a different chromatographic behavior on cIEX chromatography due to different hydrophobicity. Besides, the SDS-PAGE profiles of the cation exchange components of *P. glauca* revealed the presence of an extensively crosslinked α2-chain which could not be separated from the β22-component under these chromatographic conditions.

This is the first study providing information about differences in the subunit composition of *M. merluccius* and *P. glauca* ASC by means of a combination of GPC-LS, cIEX, and SDS-PAGE techniques.

## Supplementary Materials

The following are available online at https://www.mdpi.com/2073-4360/12/8/1734/s1, Figure S1: Gel permeation chromatograms of *M. merluccius* ASC dissolved in mobile phase and treated at different temperatures and stirring times, Figure S2: GPC of *P. glauca* ASC dissolved in mobile phase and treated at different temperatures and stirring times.
